# The Expression and Prognostic Significance of ICOS in NSCLC Integrated Pan-Cancer and Multi-Omics Analyses

**DOI:** 10.7150/ijms.93262

**Published:** 2024-03-03

**Authors:** Mengting Chen, Xiaotian Yan, Bo Hong, Yufei Xiao, Yun Qian

**Affiliations:** 1 Department of Clinical Laboratory, Stomatology Hospital, School of Stomatology, Zhejiang University School of Medicine, Zhejiang Provincial Clinical Research Center for Oral Diseases, Key Laboratory of Oral Biomedical Research of Zhejiang Province, Cancer Center of Zhejiang University, Hangzhou 310006, China.; 2 Department of Pathology, The Second Affiliated Hospital, Zhejiang University School of Medicine, Hangzhou 310009, China.; 3 Department of Clinical Laboratory, The Second Affiliated Hospital, Zhejiang University School of Medicine, Hangzhou 310009, China.

**Keywords:** ICOS, NSCLC, immune infiltration, immunohistochemistry, PD-L1, soluble ICOS/PD-L1

## Abstract

**Background:** Inducible co-stimulator (ICOS) shows great potential in the regulation of innate and adaptive immunity. However, previous studies of ICOS have often been limited to one or two levels.

**Methods:** Using the data from the online database, the immunohistochemistry, and enzyme-linked immunosorbent assays, we investigated the role of ICOS / PD-L1 on patients with NSCLC at the mRNA, protein, and serum levels.

**Results:** Our data revealed that unlike most solid tumors, the mRNA expression of *ICOS* was down-regulated in NSCLC. In addition, our data also showed that mRNA expression levels in *ICOS* are negatively associated with poor clinicopathologic grading but positively associated with better prognostic outcomes and higher Tregs infiltration level. Immunohistochemistry showed that ICOS correlated negatively with the T stage; while PD-L1 levels correlated positively with the N stage and FOXP3 levels. Serological biomarker analysis showed that patients with NSCLC had lower sICOS levels, which increased significantly post-surgery, and combined sICOS and sPD-L1 diagnosis improved efficacy and accuracy of disease diagnosis.

**Conclusion:** Our findings support that ICOS suggests lower pathological staging and better prognosis. ICOS is a potential diagnostic and prognostic biomarker for NSCLC.

## Introduction

Over the past decades, lung cancer has been the most common cancer worldwide. It is usually detected at an advanced stage because it is initially asymptomatic, resulting in the highest mortality rate of all common cancers 1. According to global cancer statistics, it is estimated that more than 2 million individuals are newly diagnosed with lung cancer annually 2. Non-small-cell lung cancer (NSCLC) accounts for approximately 85% of primary lung cancers, including the most common subtypes: Lung adenocarcinoma (LUAD) and lung squamous cell carcinoma (LUSC) 3. Although most patients with NSCLC receive multiple conventional therapies, including surgery, radiation, chemotherapy, and targeted therapy 4, the overall cure and survival rates for patients remain low 5. Therefore, it is necessary to diagnose NSCLC in an early stage and evaluate the expression of immune molecules and their clinical significance in patients with NSCLC, which might help to guide the selection and improvement of effective immunotherapy.

Inducible T cell costimulator (ICOS), a member of the CD28/B7 superfamily, conveys a positive co-stimulatory signal to activated T cells upon binding to its ligand (ICOS-L) 6. ICOS has dual effects in tumor advancement: On the one hand, ICOS can promote the differentiation of effector T helper (Th) cells and enhances the secretion of cytokines, such as human interleukin (IL)-10, IL-17, and interferon gamma (IFN-γ), to enhance the anti-tumor T cell response; on the other hand, ICOS promotes regulatory T cells (Tregs), which support tumor development 7, 8. Therefore, the expression of ICOS in many series has a variable impact on prognosis depending on the type of cancer 9. The expression of ICOS by tumor microenvironment (TME) Tregs is associated with poor prognosis in many solid cancers, such as melanoma 9, breast cancer 10 and gastric cancer 11. Although a study reported that changes in ICOS expression correlated with the clinical outcome of patients with LUAD 12, there have been few studies exploring the expression of ICOS in NSCLC.

As a major immune checkpoint for tumor-specific T cell responses, programmed death-ligand 1 (PD-L1), also known as CD274 and B7-H1, is a transmembrane protein commonly expressed on the surface of antigen-presenting cells and tumor cells, which plays an important role in downregulating T-cell activation and promoting tumor immune escape by binding to programmed cell death protein-1 (PD-1) on activated and dysfunctional T cells 13, 14. A previous study suggested that PD-L1 degradation by drug treatment effectively enhances tumor immunotherapy 15. In patients with NSCLC, increased PD-L1 expression is associated with poor prognosis 16 and the overall survival (OS) of patients might be prolonged by inhibiting the PD-1/PD-L1 signaling axis 17. This correlation emphasizes the critical role of PD-L1 in NSCLC progression and its potential as a prognostic marker for patients.

The TME, which comprises cancer cells, the intricate cytokine environment, extracellular matrix, immune cell subsets, and other components, is a dynamic system that has been intensively investigated 18. Growing evidence suggests that the characteristics of tumor-infiltrating immune cells (TIICs) in the TME correlates highly with disease aggressiveness and patient outcome in several types of cancer 12, 19. In this study, we focused on the suppressor cells that can affect tumor progression and drug efficacy. Forkhead box P3 (FOXP3) ^+^ T regulatory cells and CD163^+^ M2 macrophages are representative suppressor cells, which are associated with a poorer outcome 20, 21. However, few studies have examined the tumor immune invasive and prognostic role of TIICs in NSCLC. The use of a single biomarker to predict clinical response is limited by the complexity of the tumor immune response. Therefore, in addition to a potential role in early diagnosis and prognosis, the combination of immune molecules and TIICs in the TME can assess tumor conditions, and even guide therapeutic strategies for personalized immunotherapy of patients.

In this study, we assessed the expression patterns and clinical significance of ICOS in patients with NSCLC from the mRNA, protein, and serological aspects, as well as their correlation with PD-L1, tumor immune infiltrating cells and prognostic significance. The main aim of our research was to provide new insights into the development of clinical research and immune-targeted therapy in NSCLC.

## Materials and Methods

### ICOS expression and datasets obtained

We searched Human Protein Atlas (HPA, https://www.proteinatlas.org/) and got the ICOS RNA and protein expression summary in humans. The expression profiling data and clinical data for 33 tumors were downloaded from The Cancer Genome Atlas (TCGA, https://portal.gdc.cancer.gov/), whereas the TCGA_GTEx dataset includes TCGA samples, and normal samples were downloaded from Xena at the University of California, Santa Cruz (UCSC) (https://xenabrowser.net/datapages/) were obtained.

### Kaplan-Meier survival analysis

The Kaplan-Meier Plotter (https://kmplot.com/analysis/) is a powerful online tool to assess the impact of 54,000 genes on survival in 21 types of cancer, using more than 10,000 cancer samples, including 371 liver, 1440 gastric, 3452 lung, 2190 ovarian, and 6234 breast cancer samples 22. We analyzed the relationship between *ICOS* expression and overall survival (OS), first progression (FP), and Post-progression survival (PPS) in NSCLC. According to the median values of mRNA data, patients with NSCLC were split into the low and high *ICOS* expression groups.

### Immune infiltration analysis based on single sample gene set enrichment analysis and tumor immune estimation resource

Single-sample gene set enrichment analysis (ssGSEA) is used for immune deconvolution analysis to evaluate the abundance of immune cell types, T-cell infiltration score (TIS), Immune Infiltration Score (IIS), and fraction of immune cells (Immune Score) in a sample based on the expression level of immune cell-specific marker genes 23. We computed the enrichment scores of tumor immune cells in patients with NSCLC using ssGSEA implemented in the R package Gene set variation analysis (GSVA) from the TCGA dataset. The following 24 types of immune cells were obtained: activated dendritic cells (aDCs), B cells, CD8+ T cells, cytotoxic cells, dendritic cells (DC), eosinophils, immature dendritic cells (iDCs), macrophages, mast cells, neutrophils, natural killer (NK) CD56 bright cells, NK CD56 dim cells, NK cells, plasmacytoid dendritic cells (pDCs), T cells, Th cells, central memory T cells (Tcms), effector memory T cells (Tems), follicular helper T cells (TFHs), Tgd, type-1 T helper (Th1) cells , type-17 T helper (Th17) cells, type-2 T helper (Th2) cells, and Tregs.

Correlation analysis between gene expression and immune infiltration was performed using TIMER2.0 software (http://timer.cistrome.org/). We analyzed the correlation of *ICOS* expression levels with the infiltration level of tumor immune infiltrating cells, including Tregs and M2 macrophages, via the Gene Module of TIMER2.0 using quanTiseq and CIBERSORT algorithms.

### Patient and tissue specimens

The study investigated 72 patients diagnosed with NSCLC (Stage I-IIIA (79.17%)) who were treated at the Department of Thoracic Surgery in the Second Affiliated Hospital of Zhejiang University School of Medicine from September 2017 to October 2017. We only recruited Han Chinese patients into the group to prevent possible effects of ethnicity. The average follow-up time for our study was 53 ± 17 months. This study followed the ethical guidelines of the Declaration of Helsinki and was approved by the Ethics Committee of the Second Affiliated Hospital of Zhejiang University School of Medicine.

### Immunohistochemistry

Immunohistochemistry (IHC) was performed using the two-step Dako Envision system (Dako, Glostrup, Denmark) according to the manufacturer's instructions. Immunohistochemical staining was performed as described previously 24. The sections were incubated with a rabbit anti‑PD‑L1 monoclonal antibody (1:500 dilution; Abcam, Cambridge, MA, USA), a rabbit anti‑ICOS monoclonal antibody (1:100 dilution; Abcam), a rabbit anti-CD163 monoclonal antibody (1:500 dilution; Abcam), and a rabbit anti-FOXP3 monoclonal antibody (1:500 dilution; Abcam) at 4 °C overnight. The DAKO EnVision assay system (K5007) was used for the immunoassay. Slides were counterstained with Meyer's hematoxylin, dehydrated in gradient alcohol, and fixed in neutral resin. Negative controls were stained with phosphate-buffered saline instead of primary antibody.

### Manual quantification of the IHC results

IHC staining was evaluated independently by two pathologists who were blinded to the patients' clinical features and outcomes. Five different tumor fields selected at random from each sample to obtain agreement between the observer assessments for each specimen.

The staining signal of ICOS was evaluated according to the proportion of positive cells (0, ≤ 5%; 1, 6 -15%; 2, 16-0%; 3, 31- 50%; 4, > 50%) and the intensity of staining (0, no staining; 1, weak staining, light yellow; 2, mild staining, yellow-brown; and 3, strong staining, dark brown). The final score was calculated to determine the cut-off value for low and high expression group using the proportion of positive cells × the intensity staining 25. In this study, most of the staining results were negative, so the low expression was defined as a final score of 0 and high expression as a final score > 0. The expression of PD-L1 was evaluated using the Tumor Proportion Score (TPS) based on a previously described proportion score 26, which was classified as low (< 1%), intermediate (1%- 49%) or high (≥ 50%) expression. For statistical analysis, we considered only two groups: PD-L1 high expression (≥ 50%) and PD-L1 low expression (< 50%) groups.

The expression levels of FOXP3/CD163 were scored semi-quantitatively based on staining intensity and percentage of positive cells. For the percentage of positively stained cells, the scores were categorized from 0 to 4 (0, < 5%; 1, 5-25%; 2, 26-50%; 3, 51-75% and 4, > 75%), and the staining intensity was as mentioned above. The sum of these two indicators was used to provide the final IHC score, which was from 0 to 7. According to the IHC score, the tissue staining pattern was defined as low expression (IHC score = 0-2) or high expression (IHC score = 3-7)^21^. Images were captured using the automatic intelligent imaging system (EVOS M7000, Thermo Fisher Scientific, Waltham, MA, US).

### Measurement of sICOS and sPD-L1 levels in serum using the ELISA method

In this experiment, serum was collected from 72 patients with NSCLC before and after surgery and from 20 healthy volunteers. Blood was collected in test tubes and centrifuged at 2000 × g for 10 min at 4 °C within 30 min after collection. The liquid upper layer (serum) was stored at -80 °C. The levels of sICOS and sPD-L1 were analyzed using enzyme-linked immunosorbent assay (ELISA) kits (R&D Systems Inc., Minneapolis, MN, USA) according to the manufacturer's instructions. After incubation for 6 h, the optical density of each well was recorded to detect sICOS and sPD-L1 using a microplate reader. Each sample was analyzed in duplicate.

### Statistical analysis

Statistical Product and Service Solutions (SPSS) 26.0 statistical software was used for statistical analyses (IBM Corp., Armonk, NY, USA). The statistical comparison between clinicopathological characteristics and ICOS/ PD-L1 expression was evaluated using the chi-squared test, Fisher's exact test, and the likelihood-ratio chi-squared test. The correlation between serum sICOS and sPD-L1 was analyzed by logistic regression analysis and subject work characteristics (ROC) curves. ROC analysis was performed using binary logistic regression model predictive values of joint variables. The log-rank test was used to determine differences in OS, FP and PPS between the high and low expression groups using the Kaplan-Meier Plotter. The Mann-Whitney U test was used for the two-group comparisons of non-normally distributed (nonparametric) variables. P < 0.05 was considered statistically significant.

## Results

### The expression landscape of ICOS from the HPA database

**Figure 1:**
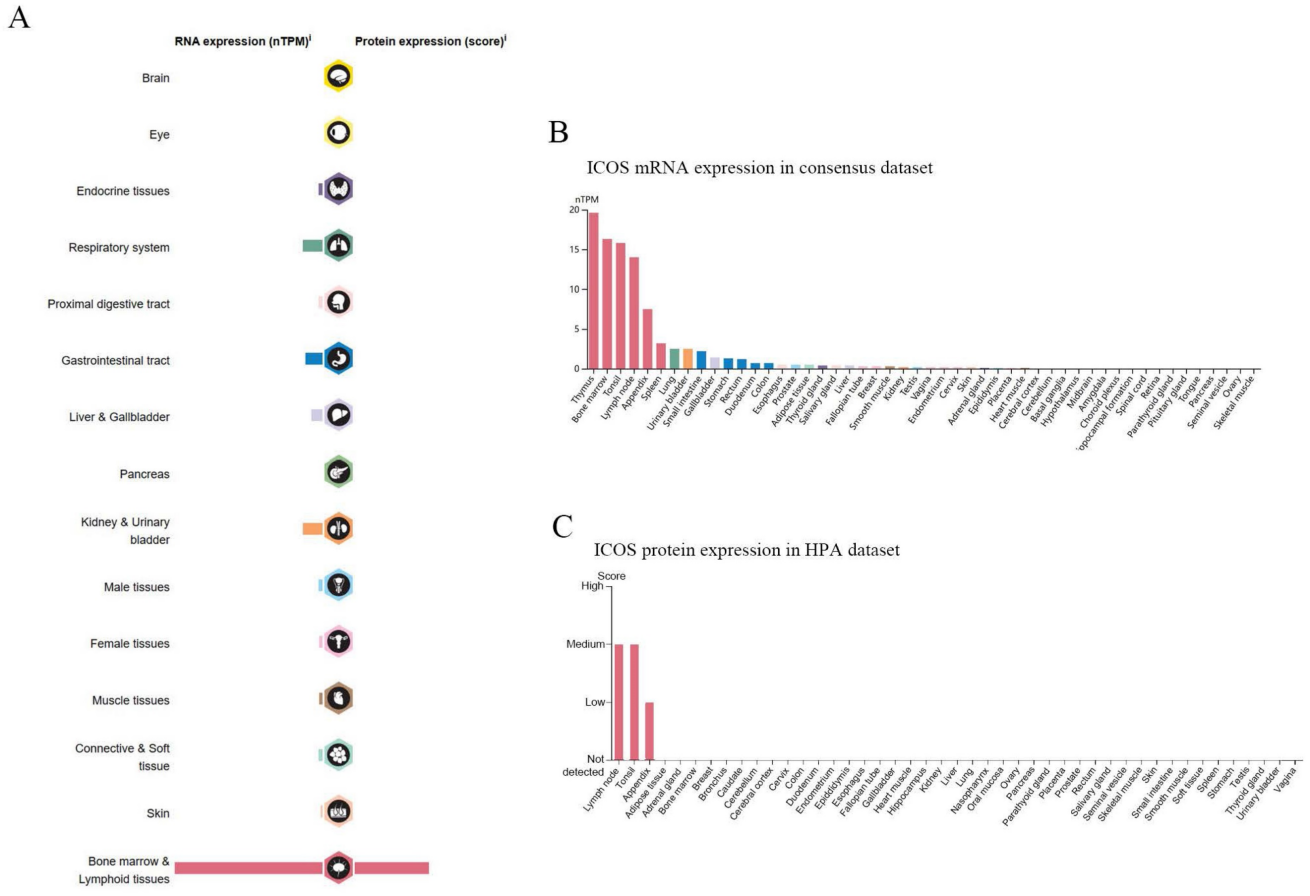
RNA and protein expression profile of ICOS in human organs and tissues. (**A**) The summary of ICOS mRNA and protein expression in human organs and tissues; (**B**) *ICOS* mRNA expression summary in different human organs and tissues based on consensus dataset; (**C**) ICOS protein expression summary in different human organs and tissues.

The mRNA of *ICOS* was widely expressed in a variety of organs and tissues (Figure 1A), most of which were expressed at low levels, but the protein expression of ICOS sites was few. As of June 2023, the HPA database already contains proteomic analyses of 27,520 antibodies against 17,288 unique proteins. A consensus dataset of samples from the HPA database showed that *ICOS* mRNAs are expressed predominantly in the thymus, bone marrow, tonsils, lymph nodes, appendix, spleen, lung, urinary bladder, small intestine, and gallbladder (Figure 1B). However, the protein of ICOS was only expressed in a few tissues such as the lymph node, tonsil, and appendix (Figure 1C).

### The expression of *ICOS* in pan-cancer from the TCGA database

**Figure 2:**
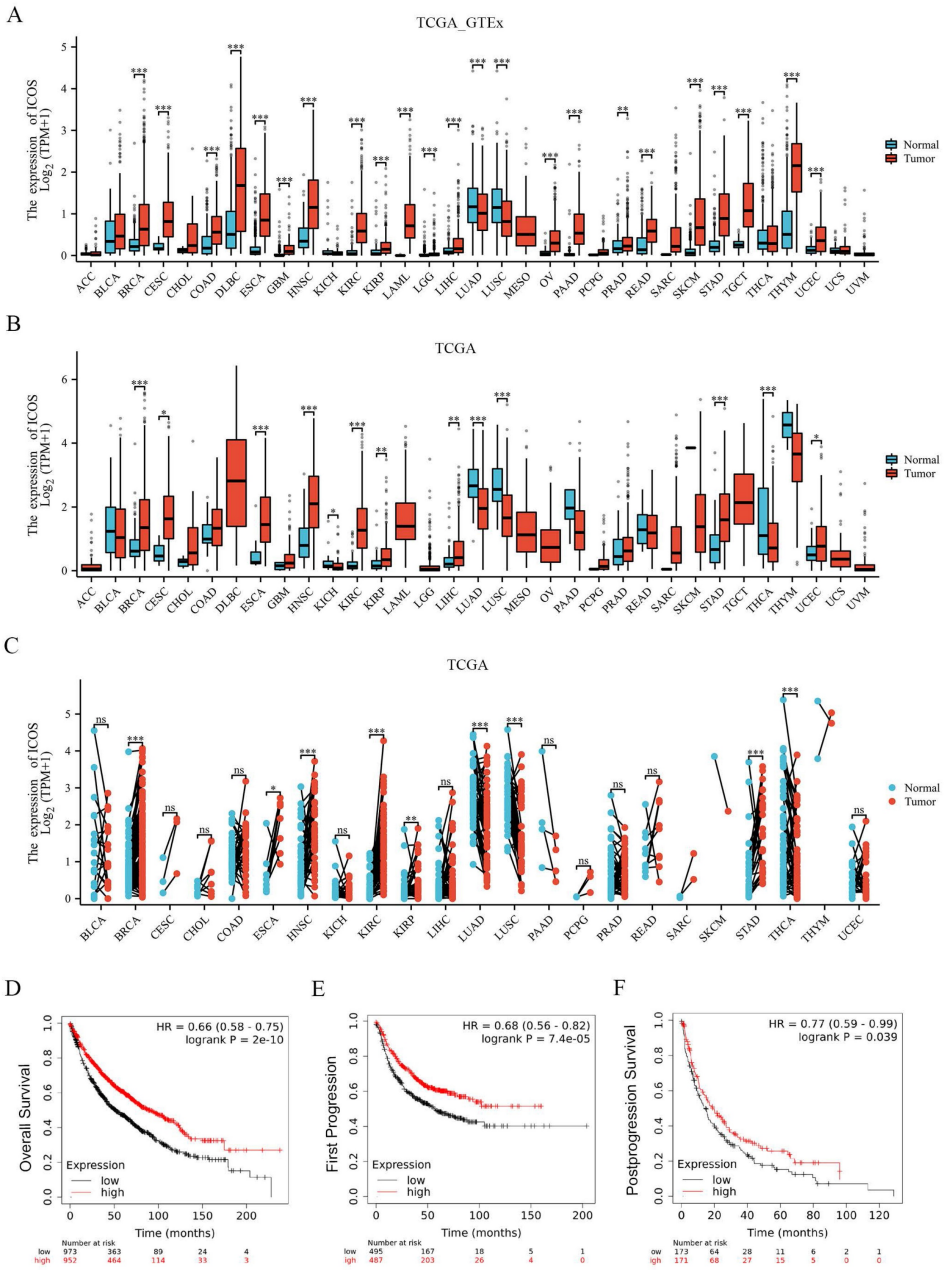
The expression of *ICOS* mRNA in pan-cancer, and the prognostic value of *ICOS* in NSCLC based on Kaplan-Meier plotter-determined survival. (**A**) Expression of *ICOS* between the 33 cancers and normal tissues in unpaired sample analysis; (**B**) Expression of *ICOS* between the 33 cancers and paracancerous tissues in unpaired sample analysis; (**C**) Expression of* ICOS* in paired samples of 23 tumors in TCGA database. ACC, adrenocortical carcinoma; BLCA, bladder urothelial carcinoma; BRCA, breast invasive carcinoma; CESC, cervical and endocervical cancers; CHOL, cholangiocarcinoma; COAD, colon adenocarcinoma; DLBC, lymphoid neoplasm diffuse large B-cell lymphoma; ESCA, esophageal carcinoma; GBM, glioblastoma multiforme; HNSC, head and neck squamous cell carcinoma; KICH, kidney chromophobe; KIRC, kidney renal clear cell carcinoma; KIRP, kidney renal papillary cell carcinoma; LAML, acute myeloid leukemia; LGG, brain lower grade glioma; LIHC, liver hepatocellular carcinoma; LUAD, lung adenocarcinoma; LUSC, lung squamous cell carcinoma; MESO, mesothelioma; OV, ovarian serous cystadenocarcinoma; PAAD, pancreatic adenocarcinoma; PCPG, pheochromocytoma and paraganglioma; PRAD, prostate adenocarcinoma; READ, rectum adenocarcinoma; SARC, sarcoma; SKCM, skin cutaneous melanoma; STAD, stomach adenocarcinoma; STES, stomach and esophageal carcinoma; TGCT, testicular germ cell tumors; THCA, thyroid carcinoma; THYM, thymoma; UCEC, uterine corpus endometrial carcinoma; UCS, uterine carcinosarcoma; UVM, uveal melanoma. (**D**) OS survival curves of NSCLC for *ICOS* (n = 1, 925); (**E**) FP survival curves of NSCLC for *ICOS* (n=982); (**F**) PPS survival curves of NSCLC for *ICOS* (n = 344); ns, no significance; * p < 0.05, ** *p* < 0.01, *** *p* < 0.001.

The *ICOS* mRNA expression was evaluated in the 33 cancer types. As Figure 2A shows, 18,102 samples were included in the unpaired sample analysis, compared with normal samples, low *ICOS* mRNA expression was observed in LUAD, LUSC (all p < 0.001) and high* ICOS* mRNA expression was observed in BRCA, CESC, COAD, DLBC, ESCA, GBM, HNSC, KIRC, KIRP, LAML, LGG, LIHC, OV, PAAD, READ, SKCM, STAD, TGCT, THYM, UCEC (all p < 0.001), and PRAD (p < 0.01). MESO and UVM could not be analyzed due to the lack of sufficient normal samples. Compared to paracancerous tissue,* ICOS* mRNA expressed significantly higher in BRCA, ESCA, HNSC, KIRC, STAD (all p < 0.001), CESC (p < 0.05), KIRP (p < 0.01), LIHC (p < 0.01) and UCEC (p < 0.05), and significantly lower in KICH (p < 0.05), LUAD (p < 0.001), LUSC (p < 0.001) and THCA (p < 0.001). This paired analysis included 11,123 samples (Figure 2B). ACC, DLBC, LAML, LGG, MESO, OV, TGCT, UCS, and UVM could not be analyzed due to the lack of sufficient paracancerous samples. Among the paired sample analyses that was performed with 11,123 samples in 23 cancers, *ICOS* mRNA expression was increased in BRCA, HNSC, KIRC, STAD (all p < 0.001) and ESCA (p < 0.05) and KIRP (p < 0.01). It was decreased in LUAD, LUSC and THCA (p < 0.001) (Figure 2C). Thus, our results from TCGA and GTEx show that its expression was significantly higher in most cancers than in normal tissues. However, there was a trend of downregulation in LUAD and LUSC, which are the two main subtypes of NSCLC; therefore, we subsequently investigated the effect of ICOS on NSCLC patients.

### Expression level of *ICOS* and prognosis of patients with NSCLC

To further confirm the importance of ICOS in the survival of patients with NSCLC, we analyzed the data using the Kaplan-Meier Plotter database. K-M survival analysis showed that ICOS mRNA expression levels were related to the prognosis of patients with NSCLC. High ICOS expression was associated with significantly longer OS, FP, and PPS compared to patients with low ICOS expression (p < 0.001, p < 0.001, p < 0.05; **Figures 2D - F**), suggesting that low ICOS expression is an indicator of poor prognosis.

### Correlation between *ICOS* mRNA expression and clinicopathological characteristics of patients with NSCLC from the TCGA database

Patients were divided into two groups based on their median expression ICOS. Patients with low expression of ICOS had a more advanced level of T stage NSCLC compared with patients with high expression of ICOS (p < 0.001, **Table 1**), and the same result was shown for clinicopathological stage (p = 0.004, **Table 1**). In addition, patients with high ICOS expression showed better treatment outcomes compared with those in the ICOS low expression group (p = 0.015, **Table 1**). In patients with NSCLC, the expression of ICOS was higher in female patients (p < 0.001, **Table 1**). However, the expression of ICOS was lower in patients who had a history of smoking (p = 0.003, **Table 1**). The expression of ICOS did not correlate significantly with other clinical features (p > 0.05, **Table 1**).

### Relationship between *ICOS* mRNA expression and tumor-infiltrating immune cells

**Figure 3:**
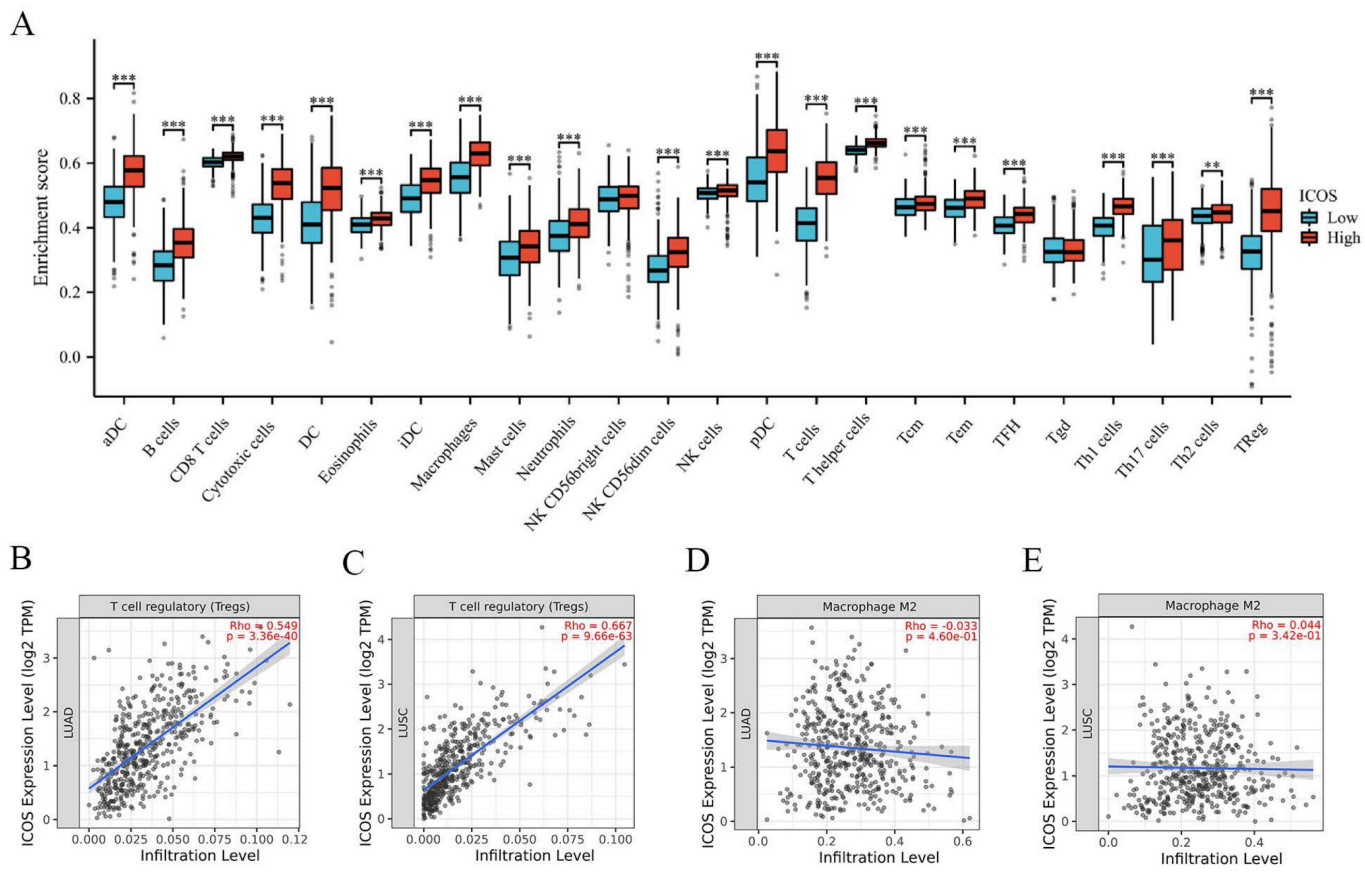
The correlation between *ICOS* mRNA expression and tumor-infiltrating immune cells. (**A**) *ICOS* were significantly different in NSCLC. A positive correlation between* ICOS* expression and the number of infiltrating Tregs in (**B**) LUAD, (**C**) LUSC. The relationship between *ICOS* expression and M2 macrophage numbers was not significant in (**D**) LUAD, (**E**) LUSC. ns, no significance; * p < 0.05, ** *p* < 0.01, *** *p* < 0.001.

Patients were divided into two groups based on their median expression of ICOS. **Figure 3A** shows that the patients with high ICOS expression had higher numbers of aDCs, B cells, CD8 T cells, cytotoxic cells, DCs, eosinophils, iDCs, macrophages, mast cells, neutrophils, NK CD56dim cells, NK cells, pDCs, T cells, T helper cells, Tcms, Tems, TFHs, Tgds, Th1 cells, Th17 cells, Th2 cells, and Tregs (p < 0.05). We also analyzed suppressive immune infiltrating cells, including Tregs and M2 macrophages. Tregs displayed a strong correlation with ICOS expression in both cohorts (r = 0.549, p = 3.36e-40 in LUAD; r = 0.667, p = 9.96e-63 in LUSC) (**Figures 3B-C**). However, the correlation between M2 macrophages and ICOS was not significant (**Figures 3D-E**).

### The expression patterns of PD-L1, ICOS, CD163, and FOXP3 in NSCLC clinical tissues

**Figure 4:**
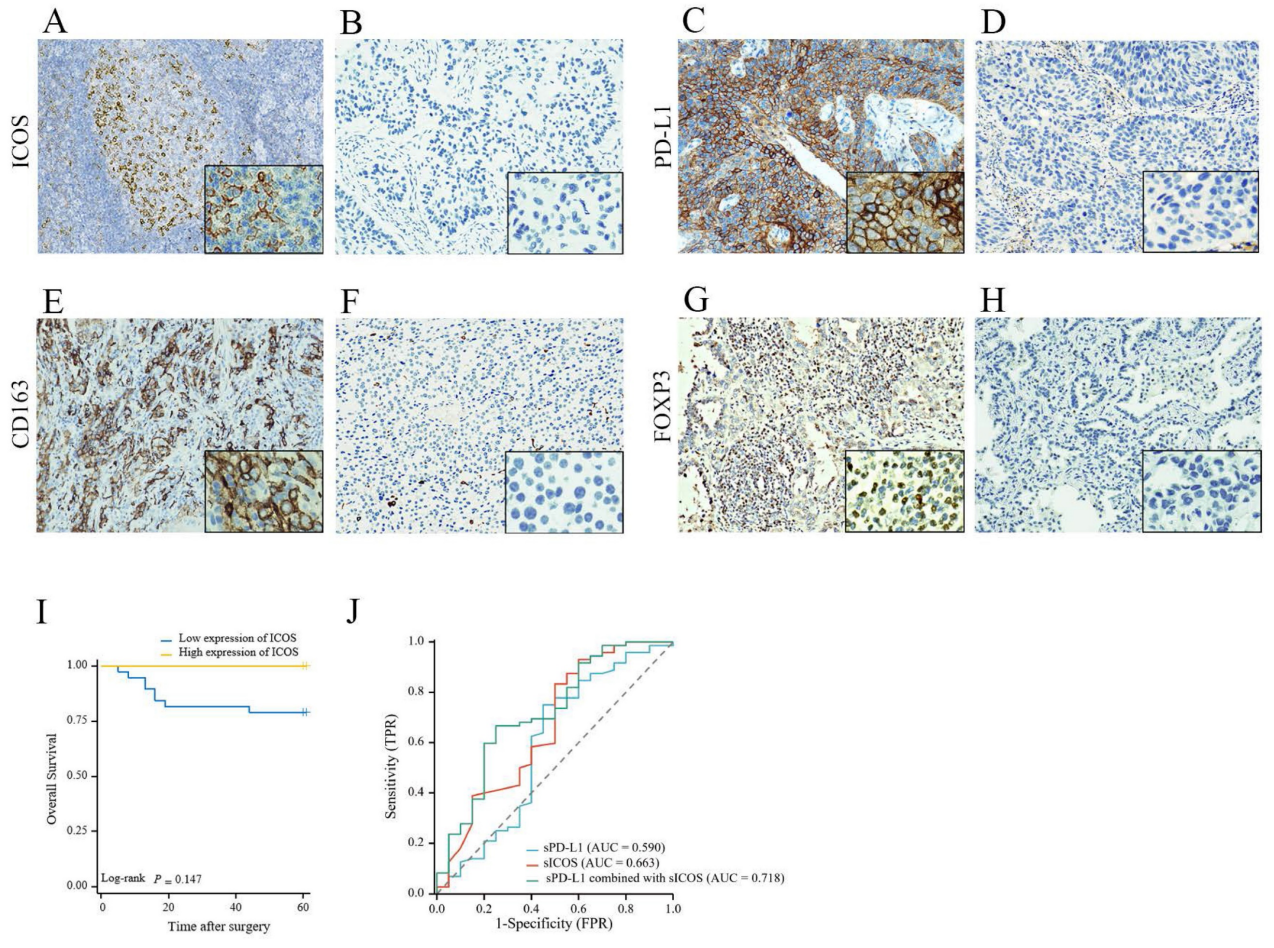
The expression level and prognosis value of ICOS, PD-L1, CD163, and FOXP3 in follow-up patients with NSCLC. Representative images of (**A**) high ICOS expression staining (staining score > 0); (**B**) low ICOS expression staining (staining score = 0); (**C**) high PD-L1 expression staining (Tumor Proportion Score ≥ 50%); (**D**) low PD-L1 expression staining (Tumor Proportion Score < 50%); (**E**) high CD163 expression staining (staining score =3-7); (**F**) low CD163 expression staining (staining score = 0-2). (**G**) high FOXP3 expression staining (staining score = 3-7); (**H**) low FOXP3 expression staining (staining score = 0-2); Original magnification ×200 and ×1000. Survival analysis of patients with high (yellow line) or low (blue line) (**I**) ICOS expression. However, no differences were observed in the follow-up patients with NSCLC. (**J**) Combined detection of sICOS and sPD-L1 in the diagnosis NSCLC. A receiver operating characteristic (ROC) curve was used to fit the combined diagnostic curve to evaluate the diagnostic value of ICOS plus PD-L1 in NSCLC, compared with ICOS alone and PD-L1 alone. The larger the area under the curve, the better the diagnostic performance. * *p* < 0.05; ** *p* < 0.01.

The median follow-up time of the retrospective analysis was 53 ± 17 (mean ± standard deviation [SD]) months (up to October 14, 2022). Lung tissue samples were obtained from 72 patients during the diagnosis process, and the characteristics of the patients are summarized in **Supplementary Table S1**. IHC was used to stain tumor tissues; however, because of the absence of some samples, ICOS levels were only determined in 47 patients, PD‑L1 levels were only measured in 62 patients, CD163 levels were only determined in 67 patients, and FOXP3 levels were only determined in 66 patients. In this study, only 19.15 % of patients expressed ICOS, i.e., most of the staining results were negative; therefore, a final score > 0 was defined as high expression. Among them, 9 patients were categorized as having high ICOS expression and 38 patients were categorized as having low ICOS expression. We found that most patients (83.87%) expressed PD-L1. Among the samples, 14 patients were categorized as having high PD-L1 expression, and 48 patients were categorized as having low PD‑L1 expression. The expression of ICOS and PD-L1 proteins on the tumor cells of patients with NSCLC was regionally distributed in most cases. Under the microscope, ICOS was mainly stained on the cell membrane; however, the cytoplasm might also be weakly pigmented, while PD-L1 could be observed in the cell membrane, cytoplasm, or both. Representative images are shown in **Figures 4A-D**.

CD163 is strongly expressed on the cell membrane of M2 macrophages and cancer cells in NSCLC 27 Almost all patient samples showed expression of CD163 (98.51%). Among them, 24 patients showed low expression and 43 patients showed high expression. FOXP3 expression mainly exhibited a mixture of diffuse staining in the cytoplasm, nucleus, or both. 81.82% of NSCLC samples expressed FOXP3 and there were 33 patients with high expression and 33 patients with low expression. Representative images are shown in **Figures 4E-H**.

### Survival analysis of patients with NSCLC in clinical cases

The OS associated with ICOS expression in NSCLC is shown in **Figure 4I**. With the day of resection as the starting point, the end of OS was defined as the day when survival or death was confirmed. Medical advances meant that as of October 14, 2022, there were only 12 deaths out of the 72 followed-up patients. Although the ICOS expression levels had no significant correlation with patient OS, we still observed that patients with low ICOS expression had a shorter OS and might have a poorer prognosis than those with high ICOS expression (p > 0.05, **Figure 4I**). Perhaps because of the small number of patients or the short follow-up period, we did not find a relationship between ICOS content and survival.

### Statistical association between ICOS / PD-L1 and clinicopathological features of patients with NSCLC in clinical cases

Patients with low ICOS expression had a more advanced T stage disease compared with patients with high ICOS expression. The expression of ICOS in patients with stage T2/T3/T4 disease was lower than in patients with T1 stage disease (p = 0.007, **Table 2**). We did not find a correlation between ICOS and N stage, M stage, clinicopathological stage, sex, age, smoking history, or prognosis.

Patients with high expression of PD-L1 had a more advanced level of NSCLC in the N stage, compared with patients with low expression of PD-L1. The expression of PD-L1 in patients with stage N1/N2/N3 disease was higher than in patients with T0 stage disease (p = 0.019, **Table 2**). We did not find a correlation between PD-L1 and T stage, M stage, clinicopathological stage, sex, age, smoking history, or prognosis.

### Correlation between PD-L1/ICOS expression and FOXP3/CD163 infiltration

In the group with high expression of PD-L1, CD163 and FOXP3 mainly showed high expression (85.71% and 78.57%, respectively). No association was found between ICOS and CD163 expression levels (p > 0.05). In the ICOS high expression group, FOXP3 expression was more inclined to be low, and the difference was statistically significant (p = 0.012, Table 3). Compared with that in the PD-L1 low expression group, the PD-L1 high expression group had higher FOXP3 expression (p = 0.033, Table 3).

### Statistical association between serum sICOS / sPD-L1 and clinicopathological features of patients with NSCLC in clinical cases

Peripheral blood biomarkers are an attractive alternative to tumor-based markers. We observed found that the level of sICOS in preoperative patients with NSCLC was significantly lower than that in the control group (p = 0.026, Table 4), while sPD-L1 levels did not differ significantly between the two groups (p > 0.05, Table 4). Then, we obtained the cut off values of sICOS and sPD-L1 based on the ROC curve and divided the cohort into four groups (Table 5). A significantly higher proportion of healthy individuals were present in the double-high expression group (p < 0.001, Table 5). In this study, postoperative sICOS and sPD-L1 of the patients were also measured. The results showed that sPD-L1 levels were not significantly different before and after surgery (p > 0.05, Table 4). Compared with the preoperative groups, sICOS levels were significantly higher in the postoperative groups (p = 0.026, Table 4).

As shown in Table 6, no significant correlation was observed between sICOS/sPD-L1 levels and T stage, N stage, M stage, clinicopathological stage, or sex in preoperative patients (p > 0.05, Table 6). However, we found that sPD-L1 levels were higher in patients older than 60 years old compared to those younger than 60 years old (p = 0.028, Table 6). Compared with those with low carcinoembryonic antigen (CEA) levels, sICOS levels were lower in preoperative patients with high CEA levels (p = 0.035, Table 6). ROC analysis (**Figure 4J**) showed that the area under the curve (AUC) for single sICOS in serum was 66.3% (AUC) (p = 0.026) and for single sPD-L1 in serum was 59.0% (p>0.05). The area under the curve for combined sICOS and sPD-L1 in serum was 71.8% (p = 0.003). Thus, discrimination became good when combining serum sICOS with sPD-L1 in the multivariate model. The AUC for the combined diagnosis was greater than for sICOS, with the difference approaching statistical significance (p = 0.198).

## Discussion

ICOS is a member of the CD28 family, a co-stimulatory receptor expressed on activated T lymphocytes 28. First, we systematically evaluated the mRNA and protein expression patterns of ICOS in different human normal tissues and various types of tumors by bioinformatics analysis. The results showed that *ICOS* expression was up-regulated in most cancer tissues. Interestingly, however, we detected a decrease in *ICOS* expression in lung cancers such as NSCLC. In order to investigate this phenomenon in depth, we comprehensively analyzed the transcriptional expression, protein level and serological characteristics of ICOS in NSCLC samples using various techniques, and compared them with PD-L1 expression and immune infiltration.

At the transcriptome level, we found that *ICOS* mRNA expression correlated inversely with the clinicopathological classification but predicted better therapeutic outcome and prognosis. These findings suggested that *ICOS* expression is not only an independent prognostic factor, but also is involved in the clinicopathological tissue progression in patients with NSCLC, which was consistent with a previous report 29.

Given that PD-L1 has been more adequate in NSCLC studies, we did not reuse data on PD-L1 from public databases in order to obtain more clinically translational results. Instead, we collected specimens from clinical NSCLC patients and examined the expression of PD-L1 from both tumor tissues and peripheral serum. Immediately after, we investigated the protein levels of PD-L1 and ICOS in 72 patients with NSCLC, confirming their localization and quantity in tumor tissues. In terms of protein expression, most patients expressed PD-L1, whereas ICOS was only expressed in a small fraction of the samples. This might reflect differences caused by the translation process of mRNA to protein or deviation caused by the immunohistochemical detection of proteins 21. In the pan-cancer analysis of ICOS expression in this study, we also found that ICOS was expressed at lower levels in lung tissues. In addition, low ICOS expression indicated high‑grade NSCLC progression in the T stage, while high PD-L1 expression indicated high-grade in the N stage, which is consistent with one report of 144 patients with lung cancer, which showed that PD-L1 expression in NSCLC was associated with a higher N stage, but not the T and M stages 30.

Treatment with anti-PD-1 and anti-PD-L1 therapies has consistently shown promising clinical benefits in NSCLC, which extended OS in clinical trials 31. Thus, the expression of PD-L1 might provide a theoretical basis for the implementation of immunotherapy to treat NSCLC. Montero et al. 32 showed that overexpression of the PD-L1 was associated with poor OS in NSCLC. Cancer cells inactivate T cells through PD-L1 expression; therefore, the higher the expression of PD-L1, the more the immune system is suppressed, which might lead to greater than expected cancer progression 33. Thus, PD-L1 expression could be associated with oncogenic signaling and its high expression might be involved in increased tumor progression. However, in a study of 482 patients, no significant association was found between PD-L1 levels and overall OS 34. The differences in PD-L1 expression levels and survival between patients may be closely related to variation between individual patients as well as the method of treatment. In addition, Brody's study did not support an association between PD-L1 expression and sex, age, smoking history, tumor histology, performance status, or pathological tumor grade 35.

Primarily because of the binary effect of ICOS in the TME, studies have attempted to determine the correlation between ICOS and malignant tumor outcomes. For patients with melanoma 9 gastric cancer 11, and glioma 36, higher ICOS expression predicted poorer survival. It has been reported that the immunosuppressive effect of ICOS can be attributed to the presence of ICOS^+^ Tregs, which account for 5-10% of all peripheral CD4^+^T cells 36. Contrary to this, Zhang et al. 37 showed that ICOS expression is associated with improved survival in nasopharyngeal carcinoma (NPC), and Zhang et al*.* 38 discovered that ICOS expression is inversely associated with the TNM stage and progression of colorectal cancer (CRC), suggesting that low ICOS expression might be a predictor of progression in patients. The elevated ICOS can induce its own anti-tumor response, not only by regulating the homeostasis of immune cells, but also by regulating the production of inflammatory cytokines, such as tumor necrosis factor alpha (TNF-α) and IFN-γ, which contribute to T cell immune function 29. It is possible that ICOS improves the prognosis of NSCLC mainly by inhibiting tumor progression and regulating autoimmunity-related signaling pathways. Therefore, ICOS can be used as a potential prognostic biomarker and immunotherapeutic target in the diagnosis and treatment of patients with NSCLC. In our study, ICOS expression levels were found to be associated with clinicopathological characteristics and could identify the stage of NSCLC.

In subsequent research, we focused on the relationship between immune molecules and TIICs in the TME of NSCLC. As a complex network formed by the interaction between the immune system and tumor cells, TME is highly correlated with the development of cancer 39. Thus, a more in-depth analysis of the TME might lead to the discovery of advanced biomarkers, which could be essential for tumor signaling and predicting patient prognosis. The numbers of 24 types of immune infiltrating cells were estimated and that there were indeed differences in the TME between the high and low *ICOS* groups.

However, different types and subtypes of immune cells play different roles in the TME, such that M1 and M2 macrophages play almost opposite roles in tumor progression 40. The former usually performs antitumor functions, including directly mediating cytotoxicity and antibody-dependent cell-mediated cytotoxicity (ADCC) to kill tumor cells; the latter can promote the occurrence and metastasis of tumor cells, inhibit the anti-tumor immune response mediated by T cells, promote tumor angiogenesis, and lead to tumor progression. Therefore, M2 macrophages were further analyzed. We also conducted an analysis of Tregs, which can promote the immune escape of tumor cells by inhibiting anti-tumor immunity 41, and high infiltration of Tregs is associated with low survival in various types of cancer 42. Our findings suggested that *ICOS* correlated positively with the number of Tregs, which might be because of ICOS expression on Tregs 36, 43; however, the relationship with M2 macrophages was not significant.

Furthermore, we used IHC to identify Tregs and M2 macrophages by choosing FOXP3 and CD163 as protein markers. Studies have shown that tumor-associated macrophages (TAMs), specifically M2 macrophages and Tregs, contribute to tumor progression and poor overall survival 44, 45. Peng et al. pointed out that FOXP3^+^ Tregs induce immunosuppression in the TME, thereby promoting tumorigenesis, progression, and metastasis in NSCLC 46. In this study, we observed that the expression of FOXP3 in the high PD-L1 expression group was higher than that in the low PD-L1 expression group. However, the combined analysis of ICOS and FOXP3 expression showed opposite results. This indicated a positive effect of ICOS in NSCLC, but a suppressive role of PD-L1 in the tumor immune microenvironment.

Finally, as important forms of ICOS and PD-L1, we examined serum sICOS and sPD-L1 levels in patients with NSCLC. The results showed that preoperative patients with NSCLC had lower levels of sICOS, which might result in a reduced ability to provide important co-stimulatory signals to enhance and maintain T-cell responses in antitumor immunity. Although studies have shown that the sPD-L1 level can be used as a potential biomarker for lung cancer screening and staging prediction 47, high levels of sPD-L1 are associated with poorer survival in patients with NSCLC 48. In the present study, sPD-L1 did not differ according to the clinicopathological classification of patients with NSCLC, which was consistent with the study of Li et al. 49. We believe that the differences between these studies might be because of the small number of patients, the heterogeneity of the patient population, and the novel use of ELISA methods to determine the soluble forms.

The AUC for the combined diagnosis of sICOS and sPD-L1 in the ROC curve was greater than that of a single indicator, demonstrating that combining multiple indicators can improve the efficacy and accuracy of disease diagnosis, establish a more optimized diagnostic model, and help clinics accurately identify patient status. This finding not only reveals the potential role of ICOS in NSCLC, but also suggests that there may be an interaction or synergistic effect between PD-L1 and ICOS in regulating tumor immune response, further emphasizing their importance in the immune microenvironment. Although PD-L1 exerts immunosuppressive effects through its interaction with PD-1 and ICOS promotes immune responses by enhancing T-cell activity, they may exhibit crossover or synergistic effects under specific conditions, such as cancer immune escape and immune checkpoint blockade therapy. For example, it has been found that bispecific antibodies against PD-L1/ICOS show promise for potential application in cancer therapy 50. Other forms of ICOS and PD-L1 (such as exosomal (exo)- ICOS and (exo)‑ PD-L1) will be explored in a future study.

In summary, ICOS expression in NSCLC exhibits distinct characteristics compared to other solid tumors. In this study, we performed comprehensive detection of ICOS expression in NSCLC patient samples on multiple levels including transcriptomics, proteomics, and peripheral blood serology. The results were analyzed with correlative examination of PD-L1 expression and immune infiltration status. This integrated approach for evaluating the dynamics of ICOS during NSCLC pathogenesis could facilitate the development of more precise and personalized immunotherapy strategies for NSCLC. Concurrent modulation of multiple immune molecules in the tumor immune microenvironment may open a new chapter of cancer immunotherapy.

There are several limitations to this study. First, this study was confined to a small cohort of cases exclusively from Asia (China), resulting in a limited sample size and an insufficient number of patients to yield conclusive data. We are planning to analyze a larger patient population in our subsequent research. Second, this study was not a prospective study; therefore, we used the archived pathological specimens, but a portion of the pathology samples in this study were missing. Third, we briefly described the role played by ICOS in patients with NSCLC; however, LUAD and LUSC are clearly different at the transcriptome level and in cellular control networks; therefore, we need to further study the different subtypes of NSCLC. Fourth, because of the improvements in medical treatment, the majority of the patients in our study were survivors, making it difficult to conduct meaningful analysis regarding ICOS as potentially valuable markers for NSCLC survival or to determine the extent of ICOS as prognostic factors. Therefore, longer follow-up might be required to assess the prognostic value of ICOS.

## Conclusions

In this study, we first performed a comprehensive pan-cancer analysis of ICOS followed by focused investigation to assess ICOS expression and clinical significance in NSCLC. Through comprehensive bioinformatics analysis, IHC and serological assessments on patients with NSCLCs, we confirmed that serum levels of sICOS can be used for early diagnosis, combined sPD-L1 and sICOS diagnosis improves efficacy and accuracy of disease diagnosis, ICOS is a potential biomarker and therapeutic target associated with the infiltration of TIICs and prognosis in patients with NSCLC. This study also provides new insights into the clinical management of patients with NSCLC.

## Supplementary Material

Supplementary table.

## Figures and Tables

**Table 1:** Correlation between *ICOS* mRNA expression with clinical characteristics of patients with NSCLC from the TCGA database

Characteristic	Low expression of *ICOS*	High expression of *ICOS*	*p*-Value
N	520	521	
T stage, n (%)			< 0.001***
T1	108 (10.40%)	182 (17.53%)	
T2/T3/T4	411 (39.60%)	337 (32.47%)	
N stage, n (%)			0.104
N0	325 (31.89%)	345 (33.86%)	
N1/N2/N3	188 (18.45%)	161 (15.80%)	
M stage, n (%)			0.475
M0	387 (47.84%)	390 (48.21%)	
M1	18 (2.22%)	14 (1.73%)	
Clinicopathological stage, n (%)			0.004**
Stage I	248 (24.10%)	293 (28.47%)	
Stage II/III/IV	267 (25.95%)	221 (21.48%)	
Primary therapy outcome, n (%)			0.015*
PD	62 (7.65%)	40 (4.94%)	
SD/PR/CR	339 (41.85%)	369 (45.56%)	
Sex, n (%)			< 0.001***
Female	169 (16.23%)	251 (24.11%)	
Male	351 (33.72%)	270 (25.94%)	
Age, n (%)			0.633
<=65	228 (22.51%)	220 (21.72%)	
>65	279 (27.54%)	286 (28.23%)	
Smoker, n (%)			0.003**
No	34 (3.35%)	61 (6.01%)	
Yes	475 (46.80%)	445 (43.84%)	

* *p* < 0.05, ** *p* < 0.01, *** *p* < 0.001. ^‡^ Note: There may be missing data in the data set, and the missing data are not displayed in the contingency table; therefore, there might be a situation that the total number of some clinical variables does not correspond to the total number of samples.

**Table 2:** Correlation between ICOS and PD-L1 protein levels with clinicopathological characteristics of patients with NSCLC in clinical cases.

	Low expression of ICOS	High expression of ICOS	*p*-Value	Low expression of PD-L1	High expression of PD-L1	*p*-Value
N	38	9		48	14	
T stage, n (%)			0.007**			0.502
T1	19 (40.43%)	9 (19.15%)		26 (41.94%)	9 (14.52%)	
T2/T3/T4	19 (40.43%)	0 (0%)		22(35.48%)	5 (8.06%)	
N stage, n (%)			1.000			0.019*
N0	28 (59.57%)	7 (14.89%)		39 (62.90%)	7 (11.29%)	
N1/N2/N3	10 (21.28%)	2 (4.26%)		9 (14.52%)	7 (11.29%)	
M stage, n (%)			0.323			1.000
M0	30 (63.83%)	9 (19.15%)		39 (62.90%)	12 (19.35%)	
M1	8 (17.02%)	0 (0%)		9 (14.52%)	2 (3.23%)	
Clinicopathological stage (%)			0.158			0.544
Stage I	17 (36.17%)	7 (14.89%)		25 (40.32%)	6 (9.68%)	
Stage II/III/IV	21 (44.68%)	2 (4.26%)		23 (37.10%)	8 (12.90%)	
Sex, n (%)			1.000			0.544
Female	21 (44.68%)	5 (10.64%)		23 (37.10%)	8 (12.90%)	
Male	17 (36.17%)	4 (8.51%)		25 (40.32%)	6 (9.68%)	
Age, n (%)			0.416			0.544
≤ 60	21 (44.68%)	3 (6.38%)		25 (40.32%)	6 (9.68%)	
> 60	17 (36.17%)	6 (12.77%)		23 (37.10%)	8 (12.90%)	
Smoker, n (%)			0.721			0.580
No	22 (46.81%)	4 (8.51%)		28 (45.16%)	7 (11.29%)	
Yes	16 (34.04%)	5 (10.64%)		20 (32.26%)	7 (11.29%)	
ICOS, n (%)						0.211
Low				29 (61.70%)	9 (19.15%)	
High				5 (10.64%)	4 (8.51%)	

* *p* < 0.05; ** *p* < 0.01

**Table 3:** Correlation Between ICOS/PD-L1 Expression and FOXP3/CD163 Infiltration.

Contents	Low expression of ICOS	High expression of ICOS	*p*-Value	Low expression of PD-L1	High expression of PD-L1	*p*-Value
Low expression of CD163	17 (44.74%)	1 (11.11%)	0.138	22 (45.83%)	2 (14.29%)	0.069
High expression of CD163	21 (55.26%)	8 (88.89%)		26 (54.17%)	12 (85.71%)	
						
Low expression of FOXP3	10 (26.32%)	7 (77.78%)	0.012*	28 (58.33%)	3 (21.43%)	0.033*
High expression of FOXP3	28 (73.68%)	2 (22.22%)		20 (41.67%)	11 (78.57%)	

* *p* < 0.05; ** *p* < 0.01.

**Table 4:** The expression levels of sICOS/sPD-L1 between different groups.

		n	Median	Mean	*p*-Value
sICOS levels (pg/mL)	Controls	20	246.39	1062.56	0.026*
	Preoperative	72	184.14	268.72	
sPD-L1 levels (pg/mL)	Controls	20	2043.67	2569.57	0.218
	Preoperative	72	1526.83	1848.70	
					
sICOS levels (pg/mL)	Preoperative	72	184.14	268.72	0.026*
	Postoperative	72	207.48	282.91	
sPD-L1 levels (pg/mL)	Preoperative	72	1526.83	1848.70	0.442
	Postoperative	72	1365.07	1782.95	

* *p* < 0.05; ** *p* < 0.01.

**Table 5:** Distribution of sICOS/sPD-L1 expression subgroups in the different groups.

sPD-L1/sICOS subgroup	Controls	Patients
Double-high expression	10 (50.00%)	5 (6.94%)
Double-low expression	9 (45.00%)	47 (65.28%)
High sPD-L1/Low sICOS expression	1 (5.00%)	13 (18.06%)
High sICOS/Low sPD-L1 expression	0 (0.00%)	7 (9.72%)

**Table 6:** Preoperative sICOS and sPD-L1 levels among subgroups of NSCLC.

Characteristic	sICOS levels (pg/mL), median	*p*-Value		sPD-L1 levels (pg/mL), median	*p*-Value
T stage		0.192			0.573
T1	191.92			1475.54	
T2/T3/T4	181.54			1562.34	
N stage		0.730			0.624
N0	191.92			1515.00	
N1/N2/N3	181.54			1593.09	
M stage		0.142			0.690
M0	181.54			1546.55	
M1	210.07			1274.33	
Clinicopathological stage		0.142			0.690
Stage I/II/III	181.54			1546.55	
Stage IV	210.07			1274.33	
Sex		0.084			0.532
Female	197.10			1570.22	
Male	181.54			1372.96	
Age		0.319			0.028*
≤ 60	197.10			1270.38	
> 60	176.36			1570.22	
CEA (ng/mL)		0.035*			0.807
≤ 5	194.51			1558.39	
> 5	165.98			1495.27	

* *p* < 0.05; ** *p* < 0.01.
