# Supplementary material for: The Expression and Prognostic Significance of ICOS in NSCLC Integrated Pan-Cancer and Multi-Omics Analyses
Source: Int J Med Sci. 2024 Mar 3;21(5):795–808. doi: 10.7150/ijms.93262 (PMC11008490; doi:10.7150/ijms.93262)
Supplement: Supplementary file 1 — Supplementary table. [file ijmsv21p0795s1.pdf]

Supplementary Table S1 Clinicopathological parameters of involved NSCLC patients

Characteristics	Number of patients (n = 72)	Proportion of patients/%
Age (years)		
≤ 60	42	58.33
> 60	30	41.67
Gender		
Male	35	48.61
Female	37	51.39
Smoking status		
Smoker	28	38.89
Non-smoker	44	61.11
Histology		
Adenocarcinoma	59	81.94
Squamous carcinoma	10	13.89
Others	3	4.17
TNM stage		
I–IIIA	57	79.17
IIIB–IV	15	20.83
Tumor size (cm)		
≤ 2.5	52	72.22
> 2.5	20	27.78
Tumor size status		
T1	41	56.94
T2-4	31	43.06
Lymph node status		
N0	55	76.39
N1–3	17	23.61
Distant metastasis		
M0	58	80.56
M1	14	19.44
